# Modification of elastic stable intramedullary nailing with a 3rd nail in a femoral spiral fracture model – results of biomechanical testing and a prospective clinical study

**DOI:** 10.1186/1471-2474-15-3

**Published:** 2014-01-08

**Authors:** Martin M Kaiser, Christine Stratmann, Gregor Zachert, Maaike Schulze-Hessing, Nina Gros, Rebecca Eggert, Marion Rapp

**Affiliations:** 1 Department of Pediatric Surgery, University Medical Centre Schleswig-Holstein, Campus Luebeck, Ratezburger Allee 160, 23538 Lübeck, Germany; 2 Department of Paediatric Surgery, Hospital of Kassel, Mönchebergstr. 41-43, 34125 Kassel, Germany; 3 Department of Biomechatronics and Academic Orthopedics, University of Luebeck, Ratezburger Allee 160, 23538 Lübeck, Germany

**Keywords:** Elastic stable intramedullary nailing, Biomechanical testing, Fracture, Femur, Treatment, Children, Adolescents

## Abstract

**Background:**

Elastic stable intramedullary nailing (ESIN) is the standard treatment for displaced diaphyseal femoral fractures in children. However, high complication rates (10-50%) are reported in complex fractures. This biomechanical study compares the stiffness with a 3rd nail implanted to that in the classical 2C-shaped configuration and presents the application into clinical practice.

**Methods:**

For each of the 3 configurations of ESIN-osteosynthesis with titanium nails eight composite femoral grafts (Sawbones®) with an identical spiral fracture were used: 2C configuration (2C-shaped nails, 2 × 3.5 mm), 3CM configuration (3rd nail from medial) and 3CL configuration (3rd nail from lateral). Each group underwent biomechanical testing in 4-point bending, internal/external rotation and axial compression.

**Results:**

2C and 3CM configurations showed no significant differences in this spiroid type fracture model. 3CL had a significantly higher stiffness during anterior-posterior bending, internal rotation and 9° compression than 2C, and was stiffer in the lateral-medial direction than 3CM. The 3CL was less stable during p-a bending and external rotation than both the others. As biomechanical testing showed a higher stability for the 3CL configuration in two (a-p corresponding to recurvation and 9° compression to shortening) of three directions associated with the most important clinical problems, we added a 3rd nail in ESIN-osteosynthesis for femoral fractures. 11 boys and 6 girls (2.5-15 years) were treated with modified ESIN of whom 12 were ‘3CL’; due to the individual character of the fractures 4 patients were treated with ‘3CM’ (third nail from medial) and as an exception 1 adolescent with 4 nails and one boy with plate osteosynthesis. No additional stabilizations or re-operations were necessary. All patients achieved full points in the Harris-Score at follow-up; no limb length discrepancy occurred.

**Conclusion:**

The 3CL configuration provided a significantly higher stiffness than 2C and 3CM configurations in this biomechanical model. These results were successfully transmitted into clinical practice. All children, treated by 3CL or 3CM according to the individual character of each fracture, needed no additional stabilization and had no Re-Do operations. As a consequence, at our hospital all children with femoral diaphyseal fractures with open physis are treated with this modified ESIN-technique.

## Background

In children fractures of the femoral diaphysis occur in around 2-3% of all fractures and is the second most frequent localization affecting the lower extremity. The guidelines of the German Society of Pediatric Surgery recommend elastic stable intramedullary nailing (ESIN) for displaced fractures even in complex or spiral fractures for children older than 2- to 3-years-of-age [1]. This is based upon reported rapid recovery, fast reintegration of the patients, a reduction of the possible negative effects of immobilization and few to no complications. Further on, elastic stable nails are preferred to avoid any possible damage to the open physis and the possible complication of avascular necrosis of the femoral head following the implantation of a rigid nail [2-7]. The technique mostly used is the so called 2-C-shaped configuration, in which two nails are implanted in an ascendig matter from the distal metaphysis of the femur [8-11]. On the other hand, skin problems and soft tissue irritation are well-known problems and some publications reported complications in up to 50% of cases with an increased tendency towards complex fracture types and older children weighting more than 40 kg [12-17]. As a consequence of this type of problem, other authors prefer submuscular plating [18,19] or external fixation for those instable fractures [20]. Rapp and co-workers analyzed 31 children with femoral shaft fractures treated by ESIN-osteosynthesis: 4 needed an additional cast and 8 patients required reoperation. Indications were 4 varus deformities and 4 shortenings of the fracture (“telescoping”). In 3 cases these were caused by technical errors during implantation and 5 fractures which could not sufficiently stabilized owing to technical limitations of the method itself [21]. In conclusion, shortening, recurvation and varus seem to be the most important clinical problems associated with ESIN in femoral fractures.

End caps were recommended to improve stability of the ESIN-osteosynthesis [22], but this could not be proved in our biomechanical in-vitro testing [23]. In the current study we aimed to analyze the effect of a 3rd ESIN on stiffness, which is a valid stability parameter in biomechanical testing [24,25]. Therefore in our spiral femur fracture model we compared osteosynthesis with 2C-shaped nails (2C) vs. 2C-shaped nails with the 3rd from antero-medial (3CM) and 2C-shaped nails with the 3rd from antero-lateral (3CL), respectively. If an improvement in stiffness through one or both 3 nail configurations (3CM/3CL) occurred, these modifications would be evaluated in children with complex femur fractures.

## Methods

### Methods part A: biomechanical setting

Biomechanical testing was performed using 24 synthetic composite left femoral models, 45 cm in length with a central canal diameter of 10 mm (4th generation, Sawbones®, Vashon, Washington, USA; European department in Malmö, Sweden). The biomechanical in-vitro setting is described in detail elsewhere [23,26,27]. Each mid-shaft fracture was identically sawed by Sawbones® with a fracture length of 100 mm and an identical spiral fracture from distal lateral rotating to cranial medial (AO pediatric comprehensive classification of long bone fractures: 32D51 [28]; LiLa classification for pediatric long-bone fractures: 3.2.s.3.2. [29]. Holes of 5 mm diameter were drilled 2–3 cm proximal of the virtual physis, medial and lateral at the distal femur, to serve as entry portals for the first two nails [4]. The entry point for the 3rd nail was 2.0 cm cranial ventral of the medial entry point in one configuration and ventral of the lateral in the other. Both nails of the 2C-shaped configuration were equally pre-bent to 40° [30]. The 3rd nail was inserted unbent afterwards. Retrograde intramedullary fixation was always performed by the same pediatric surgeon, specialized in pediatric traumatology.

**Figure 1:**
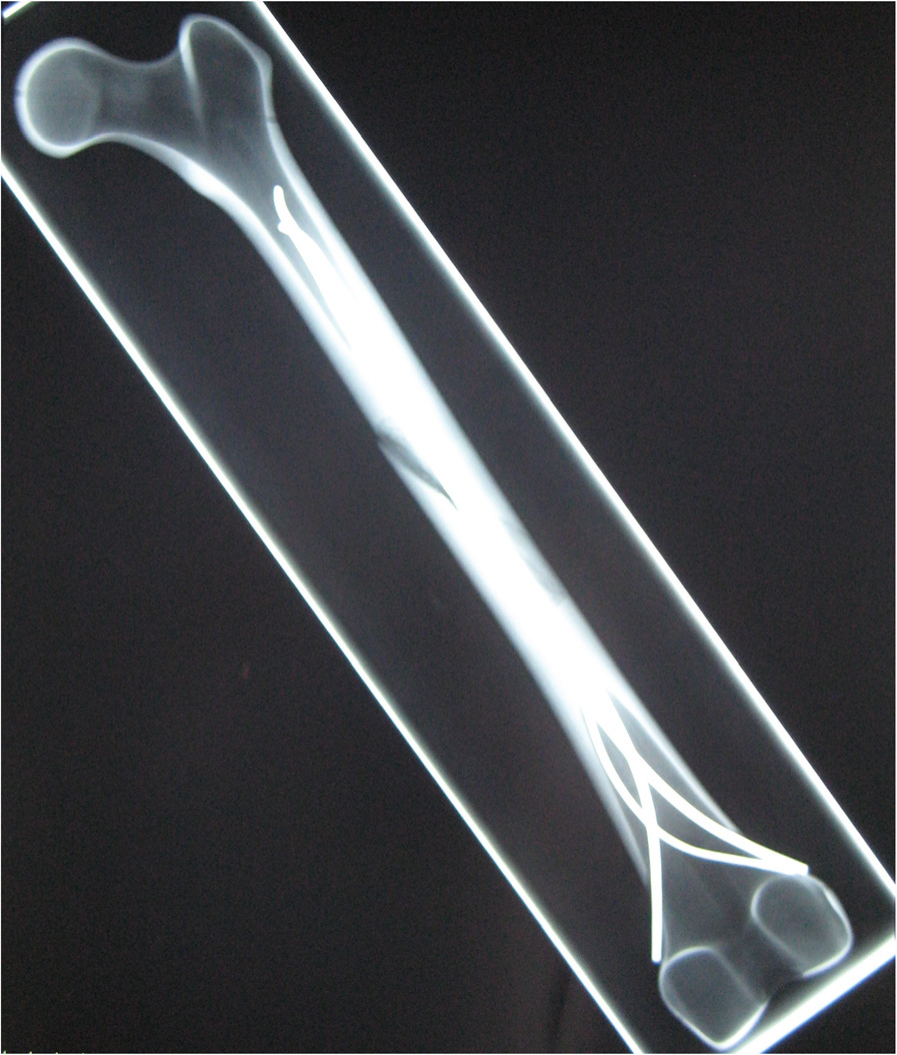
Radiograph (anterior-posterior view) of a 3CL femoral model with retrograde intramedullary nailing, showing two 3.5 mm titanium nails implanted in 2C shaped configuration and one additional 2.5 mm titanium nail implanted antero-lateral.

**Figure 2:**
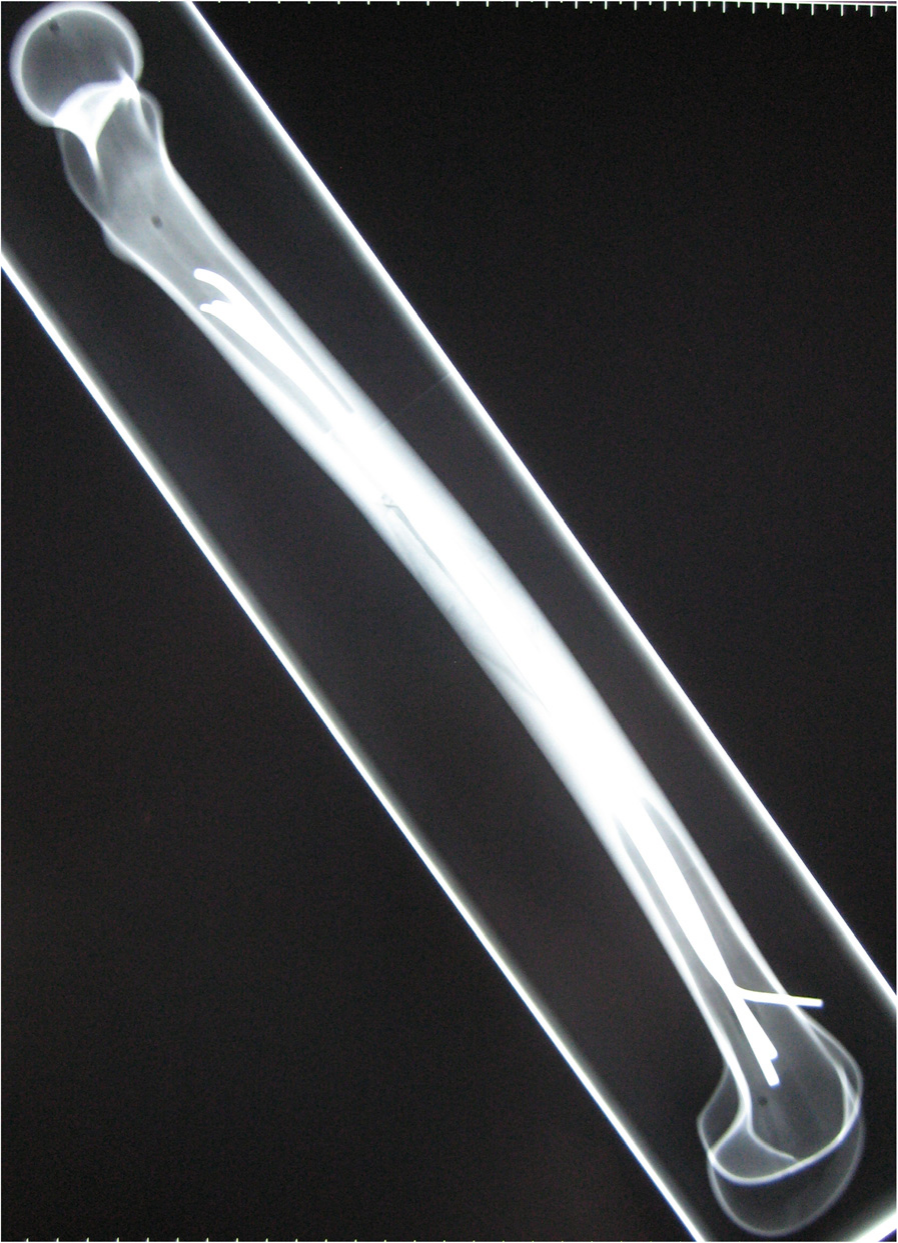
Radiograph (lateral view) of a 3CL femoral model with retrograde intramedullary nailing, showing two 3.5 mm titanium nails implanted in 2C shaped configuration and one additional 2.5 mm titanium nail implanted antero-lateral.

The 24 composite models were divided into three configuration groups:

- For the “classical” configuration with 2C-shaped ESIN pattern two 3.5 mm titanium nails were used (Group 2C: n = 8). After crossing the fracture zone the nails ended in the proximal extreme of the canal, just inferior to the greater trochanter. Fluoroscopic imaging was carried out on each specimen to confirm the correct configuration and position of the nails.

- In the modified versions with 3 nails a 2.5 mm 3rd nail was placed antero-medial (Group 3CM: n = 8) and antero-lateral (Group 3CL: n = 8, Figures 1 and 2), respectively.

A Zwick 1465 universal testing machine (UTM, Zwick GmbH & Co. KG Ulm, Germany) was used and the whole protocol was according to the ASTM F383-73 and F1264-03 standards. The femoral head and condyles were fixed in the test rig using custom-fit polymethylmethacrylate (Technovit 4006, Heraeus Kulzer, Wehrheim, Germany).

**Figure 3:**
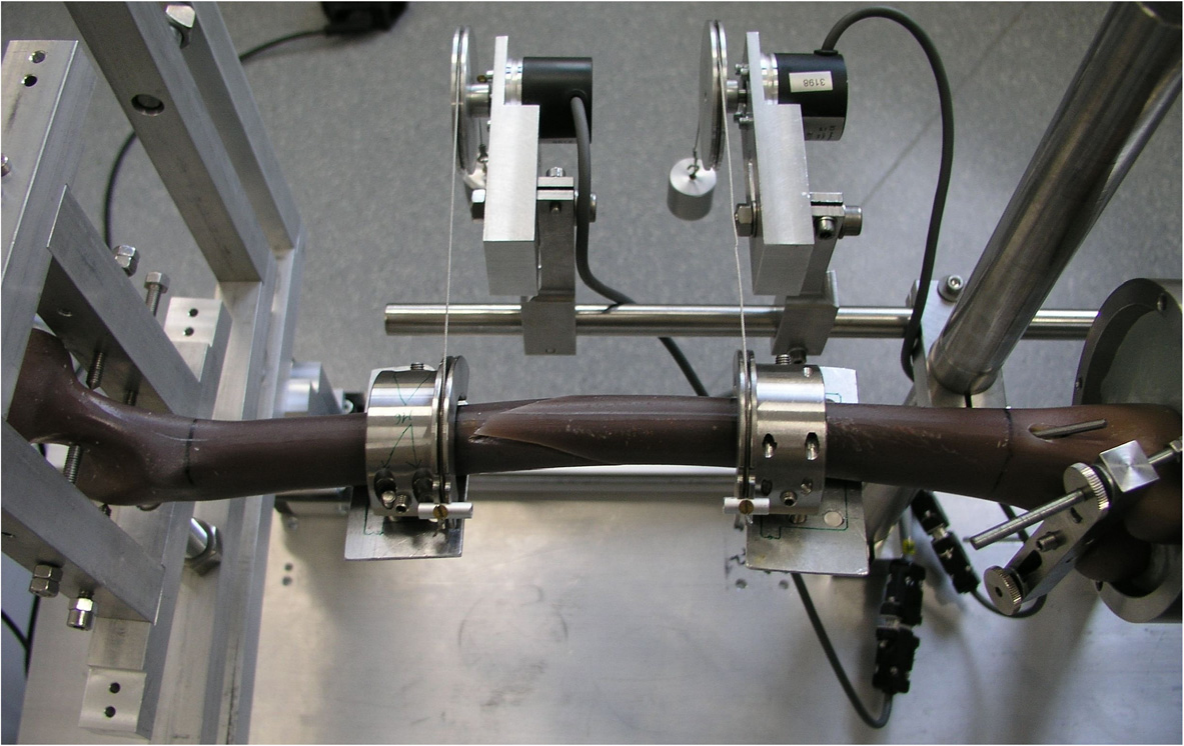
**Torsional testing of a Sawbone® with a spiral fracture.** The torsion tests were undertaken with two angular encoders at an angular speed of 20°/min; torsion was limited to 10°.

The testing of the specimens followed the standardized sequence: first 4-point bending tests [anterior–posterior (AP), posterior–anterior (PA), lateral–medial (LM) and medial–lateral (ML)], then torsion tests [internal rotation (IR) and external rotation (ER) (Figure 3)] followed by axial compression tests in the 0° (0°) and in the physiological 9° position (9°) of the femur. The *4-point bending* was determined using an incremental linear encoder (Product ID: MS30-1-LD-2, Megatron, Putzbrunn, Germany). Bending was at the midpoint of the two lower force bars with a maximum bending moment of 5 Nm and a speed of 0.05 mm/s; the maximum bend allowed was 2 mm to avoid any destruction. The *torsion tests* were undertaken with two angular encoders at an angular speed of 20°/min; torsion was limited to 10°. For the *compression* tests the femur was first positioned in the 0° position, then in the physiological 9° position of the femur with a calibrated wedge. In physiological 9° compression, the shifting at the trochanter major was analyzed corresponding to varus and valgus deformity as well the shifting at the crista intertrochanterica corresponding to ante- and recurvation. A compressive load of up to 150 N (0°) and 100 N (9°) was applied to the femoral head at a speed of 0.05 mm/s. As before, two incremental linear encoders measured the reduction of the fracture gap in the 0° and 9° positions.

The first standardized cycle was used as preconditioning; the data of the following three cycles were collected for analysis. To detect possible destructive changes to the specimens during the loading, an additional anterior-posterior stress was applied after the last individual test in the 9° position.

### Statistics for biomechanical testing

Biomechanical data (bending moments in 4-point bending, torsion stiffness in IR/ER and shortening in 0°/9°-position) were analyzed. Distributions were checked for normality (Shapiro-Wilk-Test). When a significant deviation from a normal distribution occurred, the Mann–Whitney-Test was performed. The F-Test and analyses of variance (ANOVA) were used if the distributions were normal. All values are presented as mean values. The Holm-Bonferroni correction was applied in view of the multiple series of data. Significance was set at p < 0.05.

### Methods part B: clinical setting/transfer into clinical evaluation

**Figure 4:**
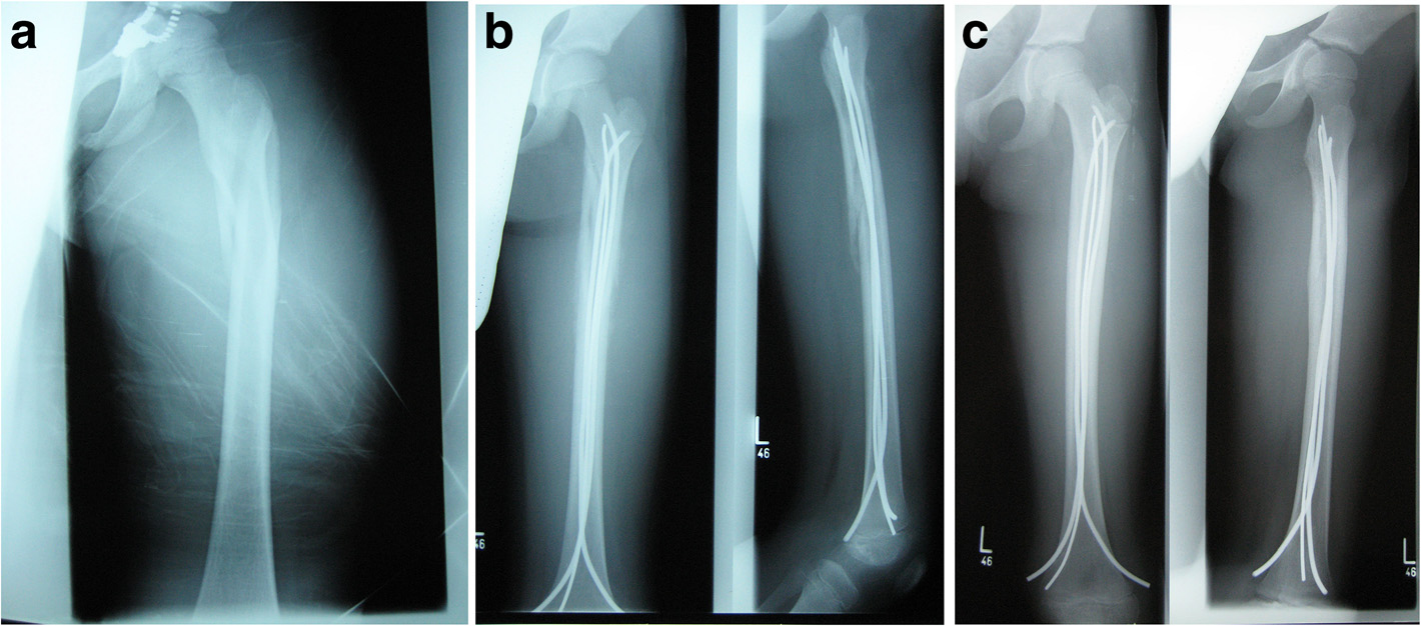
**Radiographs of a left femur with a complex proximal spiroid fracture of the shaft. a**: Fracture; **b**: X-Rays a-p and lateral after 1 month, **c**: X-Rays a-p and lateral after 4 months.

**Figure 5:**
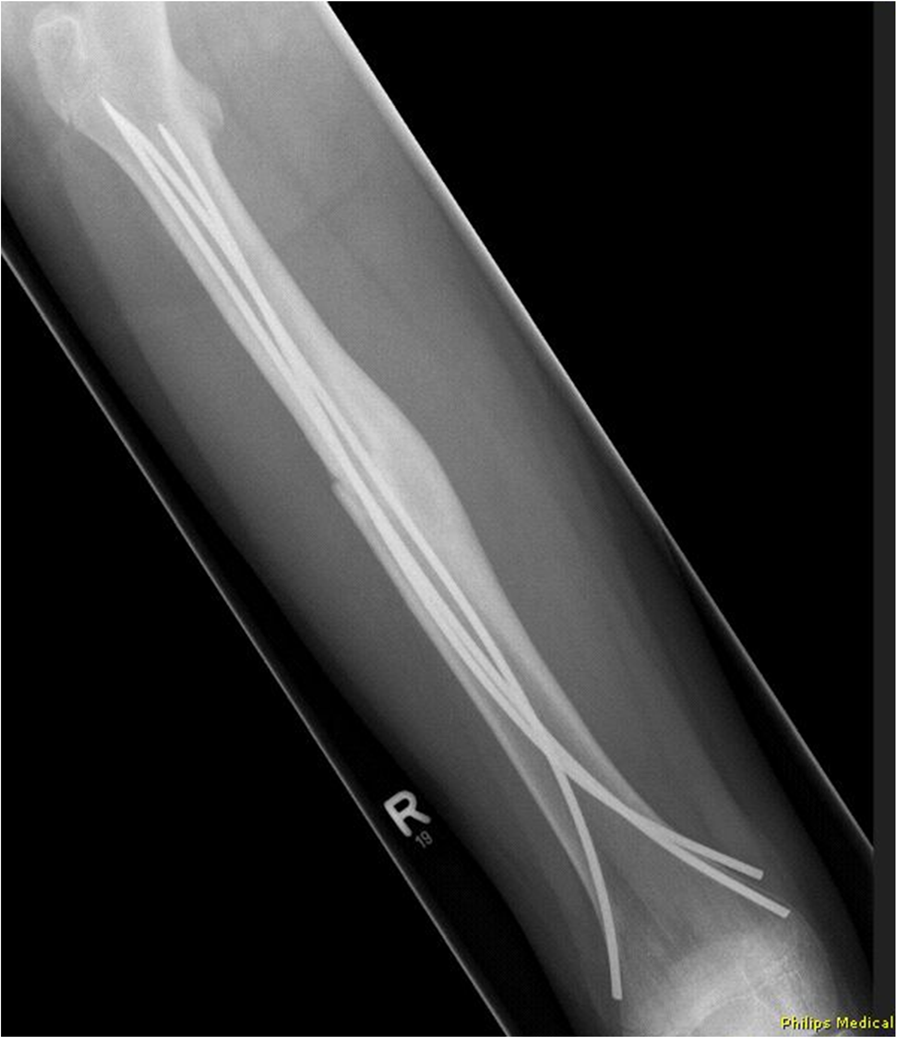
Radiograph (anterior-posterior view) of a 3CM configuration, 4 months postoperativly, diameter of the nails: 3 × 3.5 mm.

**Figure 6:**
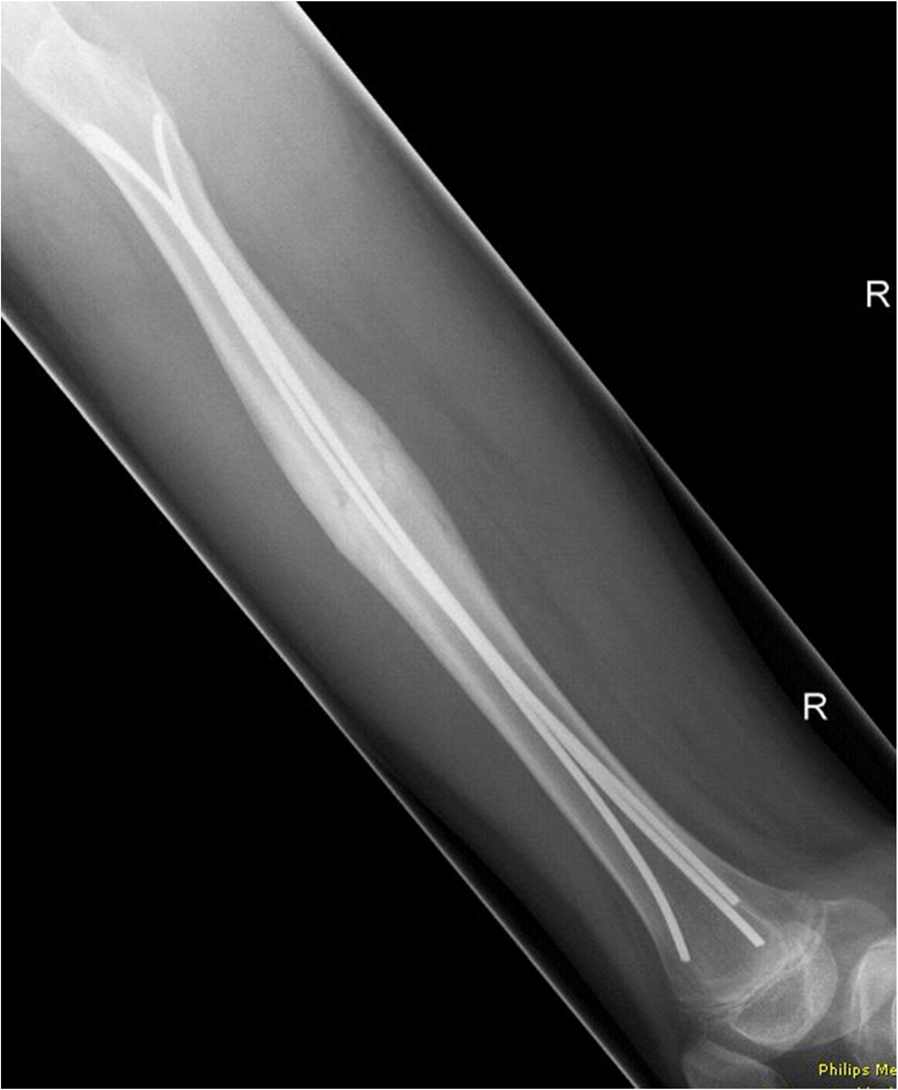
Radiograph (lateral view) of a 3CM configuration, 4 months postoperativly, diameter of the nails: 3 × 3.5 mm.

As the analysis of the biomechanical testing showed a higher stability for the 3CL configuration in two (anterior-posterior corresponding to recurvation and 9° compression to shortening/see results) of three directions associated with the most important clinical problems, we transferred this modification into clinical practice. By study protocol, first feasibility should be proved in 5 children with a either a complex/comminuted femur fracture or who weighed more than 40 kg. All 5 operations were carried out by the same pediatric surgeon, specialized in pediatric traumatology, from March 2007 onwards. After considering this modification feasible, the modified regime was carried out by other members of the staff, with the 3rd nail being placed either from antero-lateral or antero-medial (Figure 4 a-c; Figures 5 and 6) depending on the individual fracture type: having created a classical 2-C-shaped ESIN-osteosynthesis the fracture was checked for remaining instability and its tendency towards valgus (“3CM” to provide more intramedullary pressure from medial to lateral) or varus malalignment (“3CL”). The diameters of the nails were chosen based on the size of the medullar canal as revealed by X-ray. The diameters of the first 2 nails were always the same; that of the 3rd nail was chosen by reference to the configuration of the fractured bone and thus varied. Gender, age and co-morbidity of the children, nail diameter, duration of surgery, additional stabilization, re-operations, time to full weight bearing and postoperative axial deviation were all documented. Follow-up occurred after 1, 3 and 6 months then every 6 months. Limb length discrepancy, re-fractures, misalignment, range of motion and limitations in daily living were documented through clinical examination and analyzed by the modified Harris-Score [23]. Radiological examination was performed 1 day after the primary operation, and then routinely after 1 month and 3 months. If consolidation was not complete, a third X-ray was determined individually for planning implant removal.

All patients and their families gave written Informed Consent to participate in the study. The study was approved by the local ethics committee of the University of Luebeck [08–069]. ClinicalTrials.gov Identifier: NCT01673048.

## Results

### Results A: biomechanical testing

All the biomechanical results for the stiffness and the gap change during compression of the different configurations are shown in Tables 1, 2, 3. Overall, the modified 3 ESIN configurations (3CM/3CL) showed good alignment.

**Table 1:** **Comparison between stiffness of osteosynthesis with 2C-shaped nails “2C“ (Titanium Nails, Santech Nord® Company) and 2C-shaped nails with 3rd from antero-*medial*** **“3CM” (Titanium Nails, Santech Nord® Company)**

	**2C-shaped nails “2C“ (n = 8)**		**3C-shaped nails with 3rd from antero-*medial*** **“3CM” (n = 8)**	
	**Mean value (SD)**		**Mean value (SD)**	**p value**
**No statistical significant difference**				
**Anterior-posterior**	0.78 (0.29) N m/mm	~	0.66 (0.32) N m/mm	n.s.
Posterior-anterior	1.78 (1.31) N m/mm	~	2.87 (2.01) N m/mm	n.s.
Lateral-medial	0.86 (0.33) N m/mm	~	0.71 (0.23) N m/mm	n.s.
**Medial-lateral**	1.10 (0.40) N m/mm	~	1.09 (0.47) N m/mm	n.s.
External rotation	0.32 (0.18) N m/°	~	0.30 (0.13) N m/°	n.s.
Internal rotation	0.14 (0.04) N m/°	~	0.14 (0.03) N m/°	n.s.
Compression 0° (decrease in length)	0.02 (0.03) mm	~	0.02 (0.01) mm	n.s.
**Compression 9°** (decrease in length)	2.18 (2.37) mm	~	1.54 (1.24) mm	n.s.

Smaller length changes in compression tests reflect a higher stability. Special terms for biomechanical directions corresponding to the most important clinical problems in elastic stable intramedullary nailing of complex fractures: shortening (= compression 9°), recurvation (= anterior-posterior) and varus (= medial-lateral) as the most important clinical problems in elastic stable intramedullary nailing of complex fractures.

**Table 2:** **Comparison between stiffness of osteosynthesis with 2C-shaped nails “2C“ (Titanium Nails, Santech Nord® Company) and 2C-shaped nails with 3rd from antero-*lateral*** **“3CL” (Titanium Nails, Santech Nord ® Company)**

	**2C-shaped nails “2C“ (n = 8)**		**3C-shaped nails with 3rd from antero-*lateral*** **“3CL” (n = 8)**	
	**Mean value (SD)**		**Mean value (SD)**	**p value**
**3CL** ***more*** **stable than 2C**				
**Anterior-posterior**	0.78 (0.29) N m/mm	<	1.23 (0.62) N m/mm	0.007
Internal rotation	0.14 (0.04) N m/°	<	0.21 (0.07) N m/°	<0.001
**Compression 9°** (decrease in length)	2.18 (2.37) mm	>	0.61 (0.43) mm	0.023
**3CL** ***less*** **stable than 2C**				
Posterior-anterior	1.78 (1.31) N m/mm	>	1.03 (0.97) N m/mm	0.014
External rotation	0.32 (0.18) N m/°	>	0.19 (0.12) N m/°	0.004
**No statistical significant difference**				
Compression 0° (decrease in length)	0.02 (0.03) mm	~	0.03 (0.03) mm	n.s.
Lateral-medial	0.86 (0.33) N m/mm	~	0.88 (0.30) N m/mm	n.s.
**Medial - lateral**	1.10 (0.40) N m/mm	~	1.10 (0.53) N m/mm	n.s.

The Holm-Bonferroni correction was applied in view of the multiple series of data. Smaller length changes in compression tests reflect a higher stability. Special terms for biomechanical directions corresponding to the most important clinical problems in elastic stable intramedullary nailing of complex fractures: shortening (= compression 9°), recurvation (= anterior-posterior) and varus (= medial-lateral) as the most important clinical problems in elastic stable intramedullary nailing of complex fractures.

**Table 3:** **Comparison between stiffness of osteosynthesis with 2C-shaped nails with the 3rd from antero-*lateral*** **“3CL” (Titanium Nails, Santech Nord® Company) and 2C-shaped nails with the 3rd from antero-*medial*** **“3CM” (Titanium Nails, Santech Nord® Company)**

	**3C-shaped nails with 3rd from antero-*lateral*** **“3CL” (n = 8)**		**3C-shaped nails with 3rd from antero-*medial*** **“3CM” (n = 8)**	
	**Mean value (SD)**		**Mean value (SD)**	**p value**
**3CL** ***more*** **stable than 3CM**				
**Anterior-posterior**	1.23 (0.62) N m/mm	>	0.66 (0.32) N m/mm	<0.001
Lateral-medial	0.88 (0.30) N m/mm	>	0.71 (0.23) N m/mm	0.028
Internal rotation	0.21 (0.07) N m/°	>	0.14 (0.03) N m/°	<0.001
**Compression 9°** (decrease in length)	0.61 (0.43) mm	<	1.54 (1.24) mm	0.004
**3CL** ***less*** **stable than 3CM**				
Posterior-anterior	1.03 (0.97) N m/mm	<	2.87 (2.01) N m/mm	0.001
External rotation	0.19 (0.12) Nm/°	<	0.30 (0.13) N m/°	0.003
**No statistical significant difference**				
Compression 0° (decrease in length)	0.03 (0.03) mm	~	0.02 (0.01) mm	n.s
**Medial - lateral**	1.10 (0.53) N m/mm	~	1.09 (0.47) N m/mm	n.s

The Holm-Bonferroni correction was applied in view of the multiple series of data. Smaller length changes in compression tests reflect a higher stability. Special terms for biomechanical directions corresponding to the most important clinical problems in elastic stable intramedullary nailing of complex fractures: shortening (= compression 9°), recurvation (= anterior-posterior) and varus (= medial-lateral) as the most important clinical problems in elastic stable intramedullary nailing of complex fractures.

No systematic difference in stiffness was found between 2C and 3CM (Table 1). 3CL revealed a significantly higher stiffness than 2C in the anterior-posterior directions, internal rotation and also in the 9° compression, in which smaller change in length indicates higher stability (Table 2). Likewise, 3CL showed a significant higher stiffness than 3CM in the same directions: anterior-posterior, internal rotation and 9° compression and further in lateral-medial (Table 3). However, 3CL was less stable in the posterior-anterior and external rotation than 2C (Table 2) and 3CM (Table 3). 0° axial compression was not influenced by either modification.

### Results B: clinical results

We intended to treat 18 children by these 3CM or 3CL modifications (Table 4), which was possible in 16 children (10 boys, 6 girls; aged 3 to 13 years). 12 configurations were ‘3CL’ (Figure 4a-c) and 4 ‘3CM’ (Figures 5 and 6) according to the individual character of the fracture. In two patients 3CL and 3CM did not provide enough stability: Because of a contralateral tibia fracture with compartment syndrome, the femur fracture of a 15-year-old boy was fixed with four nails as this gave him the possibility of very early mobilization (No. 7). In a 3-year-old boy none of the ESIN- osteosynthesis achieved sufficient stability (No. 18). As the aim of the operation was an internal osteosynthesis an angle-stable plate osteosynthesis was performed as an exception.

**Table 4:** Data of 18 patients in whom an osteosynthesis with 3 elastic stable nails “3CM” or “3CL” was attempted

**No by random**	**Sex**	**Age**	**Type of fracture**	**Location in diaphysis**	AO-Ped-* classification	LiLa-* classification	**Size of the nails (mm)**	**3C Lateral/ medial**	**Reduction**	**Co-morbidity**	**Complication**	**Implant removal (months)**	**Axis ° (X-ray post-OP)**	
													**AP/PA**	**LM/ML**
**1**	Female	11	Oblique + wedge	Central	32D52	3.2.s.3.2	2 × 4.0 + 1 × 4.0	Lateral	Closed		No	5	0°	0°
**2**	Female	8	Comminuted	Proximal	32D53	3.2.s.4.2	2 × 3.0 + 1 × 2.0	Lateral	Closed		No	6	3° Ante	0°
**3**	Male	12	Oblique + wedge	Central	32D52	3.2.s.3.2	2 × 3.5 + 1 × 3.0	Lateral	Closed		No	3	8° Ante	12° Varus
**4**	Male	12	Oblique	Distal	32D51	3.2.s.3.2	2 × 4.0 + 1 × 3.0	Medial	Closed		No	4	0°	0°
**5**	Female	11	Comminuted, 2° open	Central	32D43	3.2.s.4.2	2 × 3.5 + 1 × 3.0	Lateral	Closed	Polytrauma	No	6	8° Ante	0°
**6**	Male	9	Transverse, 2° open	Central	32D41	3.2.s.3.2	2 × 3.5 + 1 × 3.0	Lateral	Closed	Decollement	No	5	0°	0°
**7**	Male	15	Transverse, 2° open	Central	32D42	3.2.s.3.2	2 × 4.0 + 2 × 4.0	Medial + Lateral	Closed	ContraLateral tibia fracture	No	8	0°	3°
**8**	Male	13	Transverse	Distal-metaphysis	32D41	3.2.s.3.2	2 × 3.5 + 1 × 4.0	Lateral	Closed		Skin irritation	4	0°	2°
**9**	Male	3	Long spiral	Central	32D51	3.2.s.3.2	2 × 2.5 + 1 × 2.5	Lateral	Closed		No	3	0°	0°
**10**	Male	6	Long spiral	Central	32D51	3.2.s.3.2	2 × 3.5 + 1 × 3.0	Lateral	Closed		No	5	0°	3°
**11**	Male	13	Transverse	Central	32D41	3.2.s.3.2	2 × 3.5 + 1 × 3.5	Medial	Open		no	8	0°	3°
**12**	Female	3	Long spiral	Central	32D51	3.2.s.3.2	2 × 2.5 + 1 × 2.5	Lateral	Closed		Perforation of a nail-tip	2	0°	0°
**13**	Male	2.5	Long spiral	Central	32D51	3.2.s.3.2	2 × 2.5 + 1 × 2.5	Medial	Closed		No	4	0°	7° Valgus
**14**	Female	6	Spiral	Central	32D51	3.2.s.3.2	2 × 2.5 + 1 × 2.5	Medial	Closed		No	4	0°	0°
**15**	Female	8	Oblique + wedge	Central	32D52	3.2.s.3.2	2 × 3.0 + 1 × 2.5	Lateral	Closed	Polytrauma	No	4	5° Ante	0°
**16**	Male	8	Transverse	Central	32D41	3.2.s.3.2	2 × 3.0 + 1 × 3.0	Lateral	Open		No	6	12° Ante	0°
**17**	Male	3	Long spiral	Central	32D51	3.2.s.3.2	2 × 2.5 + 1 × 2.5	Lateral	Closed		No	3	2° Ante	0°
**18**	Male	3	Long spiral	Central	32D51	3.2.s.3.2	2 × 2.5 + 1 × 2.5	Not possible	Open		Plate osteosynthesis	3	0°	0°

*AO-Ped Classification **=** AO pediatric comprehensive classification of long bone fractures [20].

*LiLa Classification **=** LiLa classification for pediatric long-bone fractures [21].

Ante = Antecurvation, AP = anterior-posterior, PA = posterior-anterior, LM = latermal-medial, ML = medial-Lateral.

The overall duration of surgery was 45 to 120 minutes (including all soft tissue repairs and additional procedures especially in polytrauma-patients), of which 8 to 20 minutes were for the implantation of the 3rd nail. Twice an open reduction was necessary. None of the fractures required additional stabilization. Postoperative femoral antecurvation varied between 0 and 12° (mean 2.3°); varus deformity ranged from 0 to 12° (mean 1°) and valgus from 0 to 7° (mean 1°). There was no case of recurvation or “telescoping” of a fracture. In one child the ends of the nails were left too long: skin irritation prompted their removal after 4 months without further sequelae (No. 8). In another patient the tip of the nail perforated the corticalis proximal without further consequences (No. 12).

No early or late reoperations due to the femur fractures occurred in any of patients. Mobilization and full weight bearing was achieved after 6 weeks at the latest. Following implant removal after complete healing of the fractures (3–8 months after accident, mean 5 months), all patients reached the maximum Harris Score of 100 points. There were no problems after implant removal. At later follow-up (1–4 years) no limb length discrepancy occurred in any of the patients.

## Discussion

Elastic stable intramedullary nailing (ESIN) for displaced pediatric femoral fractures has become the treatment of choice for children [1]. Nevertheless complications of the ESIN-osteosynthesis occur due to technical errors during application and limitations of the method itself [12-17,30-32]. As a conclusion of literature and own data, most frequent postoperative complications seem to be shortening (“telescoping”), recurvation and varus deformation resulting in postoperative misalignment, re-do operations and the additional application of a cast or external fixation [33,34]. As standardized biomechanical studies are known to be an important precursor to clinical studies, we were sure that further experiments in this area would increase our understanding of ESIN-osteosynthesis. Thus, an adolescent-sized femur model with a standardized spiral fracture was used, because the primary targets were complex fractures in older children or weighing more than 40 kg. Because of the complexity of long spiral fractures, testing of all relevant variables was performed: 4-point bending (corresponding to deformation forces as antecurvation, recurvation, valgus and varus), external and internal rotation as well as axial 0° and physiological 9° compression (corresponding to shortening or “telescoping”).

Few reports of further biomechanical testing of the ESIN-osteosynthesis could be found; most of these where not standardized, depicted a small number of cases [35,36], evaluated only a few selected planes [35,37,38] or studied exclusively transverse and oblique fractures [36]. Benz and co-workers investigated changes of stability during 4-point-bending and torsion, but they only achieved sufficient 2-nail flexible intramedullary fixation for testing in 3 out of 10 canine bones [39].

Through our biomechanical model we compared the “classical” 2C configuration (2C-shaped 3.5 mm titanium nails) with 2 modified versions, each with an additional 2.5 mm titanium nail placed either from antero-medial (3CM) or antero-lateral (3CL). All the specimens with 3 ESIN achieved a better alignment and macroscopic stiffness of the fracture. In our specific spiral fracture type with rotation from distal lateral to cranial medial, the 3rd nail from anterior-lateral was effective in reducing 2 of the 3 causes of poor clinical outcomes mentioned above: the danger of shortening (better results in 9° compression) and recurvation (better results in anterior-posterior). That position of the 3rd intramedullary nail provided more stability for this specific type of the fracture which also might explain why contradictory results occurred in posterior-anterior versus anterior-posterior and internal versus external rotation. The further clinical problem, the valgus deviation, was not influenced by either modified configuration.

Nevertheless variations in the course of the 3rd nail might influence this system: If the planes between the 3rd nail and each of the other 2 were almost parallel, the increase in stability would barely be noticeable. On the other hand, positioning the 3rd nail in an almost equilateral triangle with the first 2 nails could result in significantly increased stability. Further CT-imaging studies with our 3 nail specimen will test this hypothesis.

A limitation of this first biomechanical study on the use of 3 nails is that a synthetic bone model was used, which cannot precisely reproduce all in-vivo conditions. However, because of the high repeatability due to minimal inter-individual variability [40-42], it was used successfully in previous biomechanical studies [23,26,27]. Furthermore, due to the configuration of the synthetic bone model, the nails could not be placed as proximally as is ideal in real operations. On the other hand, the planned configuration of the nails can be achieved more precisely in an experimental setup with an identical surgical technique, an exact pre-bending and an even introduction of the nails, than in a real surgical situation.

In spite of these limitations of the biomechanical study, our results were transferred successfully to a clinical setting. Osteosynthesis with 3 nails was first performed by a pediatric surgeon, specialized in pediatric traumatology (MMK) and then transferred to other members of the pediatric surgery department. Although the 3CL-configuration was initially preferred, the position of the 3rd nail was later chosen in accordance with the plane which was assumed to be the least stable. No clinically relevant deviation of axis or shortening was documented in osteosynthesis with 3 nails in 16 patients. None of the fractures required additional stabilization or re-operation. In 2 patients the 3 ESIN-osteosynthesis did not gain enough stability: Because of a contra lateral fracture of the tibia, a modification with 4 nails was chosen to reach early mobilization with weight bearing (No. 7) and in one long spiral fracture an angle-stable osteosynthesis had to be performed (No. 18). All patients reached the maximum Harris score of 100 points in the follow-up evaluations.

This transfer of biomechanical results into clinical practice demonstrates the following:

a) a 3rd nail, additionally implanted to a technically perfect 2C-ESIN configuration, can increase the stability of the fixation in a femoral fracture,

b) the implantation and the pre-bending of the 3rd nail must be appropriate to the individual character of the fracture and

c) despite these very good results with the 3CL or 3CM configurations not all problems can be solved with these modifications.

In essence, the fixation of complex femur fractures remains technically demanding and this comparison shows the high relevance of pre-clinical experimental studies. A high success rate can be achieved using insights gained from in-vitro studies, thus preventing children and adolescents from undergoing unnecessary clinical trials. We can recommend the use of a 3 ESIN-osteosynthesis in pediatric femur fractures, as long as the biomechanical characteristics of the fracture type are taken into account in the placement of the 3rd nail.

## Conclusion

In this biomechanical model the 3rd nail from antero-lateral ‘3CL’ was effective in reducing two of the three causes of poor clinical outcomes in ESIN-osteosynthesis: the danger of shortening (significantly higher stiffness in 9° compression) and recurvation (significantly higher stiffness in anterior-posterior bending). These in-vitro results were successfully transmitted into clinical practice and adapted (3rd nail from antero-lateral or -medial) to the individual character of each femur shaft fracture. Through the successful transmission of our biomechanical results into a clinical setting we are able to recommend the use of a 3rd nail in ESIN in pediatric femur fractures to improve the stability of the osteosynthesis and to reduce peri- and postoperative complications. As a consequence, at our hospital all femoral diaphyseal fractures in children/adolescents with open physis are treated with this modified ESIN-technique. However, a perfect technique while implanting the first two nails is always necessary.

## Competing interest

All authors declare that no benefits in any form have been received or will be received from a commercial party related directly or indirectly to the subject of this article. There is no conflict of interest for any author.

The elastic stable nails were sponsored by Santech Nord Company, Schneverdingen, Germany.

## Authors’ contributions

CS wrote the manuscript and did the biomechanical testing with support of RE, MSH, NG, GZ was responsible for all testing in the laboratory and edited the manuscript. MR organizes the research group and was responsible for the follow-up of the patients and for translation and proof-reading of all versions of the manuscript. MMK did most of the operations on the patients, revised the manuscript and all revisions, is the responsible author and the head of the study group. All authors read and approved the final version of the manuscript.

## Pre-publication history

The pre-publication history for this paper can be accessed here:

http://www.biomedcentral.com/1471-2474/15/3/prepub
